# Editorial: Women in AI medicine and public health 2022

**DOI:** 10.3389/fdata.2023.1303367

**Published:** 2023-10-13

**Authors:** Lin-Ching Chang, Anastasia Angelopoulou

**Affiliations:** ^1^Department of Electrical Engineering and Computer Science, School of Engineering, The Catholic University of America, Washington, DC, United States; ^2^School of Computer Science and Engineering, University of Westminster, London, United Kingdom

**Keywords:** AI, machine learning, neural network, medicine, public health

The landscape of technology has undergone a dramatic transformation with the widespread adoption and rapid advancement of artificial intelligence (AI). This evolution has had a profound impact on various sectors, reshaping not only the way industries operate but also fundamentally altering the way we perceive and interact with the world around us. In particular, AI are revolutionizing the fields of medicine and public health by offering innovative ways to analyze data, make predictions, improve patient care, and even playing central role in advancing agendas of inclusion and equality. However, gender disparity is still evident within the realms of AI, especially within the context of medicine and public health. Despite their accomplishments, women scientists continue to face gender-specific hurdles, such as navigating their public presence and cultivating secure, inclusive work environments.

This Research Topic from Frontiers in Big Data aims to promote and highlight the research work of women scientists, across the fields of AI in medicine and public health. This Research Topic is part of the Women in Artificial Intelligence series. In each work, the first author or the last author needs to be a woman researcher. Each paper underwent a rigorous review process, involving at least two reviewers and two rounds of thorough revisions before acceptance. Six articles were selected that comprise four original research, one brief research report, and one study protocol. Listed below are the papers that made important contributions to this Research Topic.

Kaushik et al. assessed the effectiveness of various statistical, neural, and ensemble methods for predicting weekly healthcare expenditures on two pain medications. Their analysis includes two statistical models (persistence and ARIMA), two neural network models (MLP and LSTM), and an ensemble model that combines ARIMA, MLP, and LSTM predictions. They reported that the ensemble model consistently outperformed the individual models for both medications. Their findings highlight the value of employing diverse modeling techniques for healthcare expenditure time-series forecasting.

Chen et al. conducted a study to explore the risk of stroke among women aged 65 years and older who has chronic obstructive pulmonary disease (COPD), with or without influenza vaccination. Their findings indicate that influenza vaccination was linked to a considerably lower risk of ischemic, hemorrhagic, and undefined stroke in women with COPD. Furthermore, the strength of this association appeared to increase with the frequency of vaccination. For women with a CHA2DS2-VASc score of 2 or higher, the association between vaccination and a reduced risk of hemorrhagic stroke was not as substantial as ischemic stroke. However, the authors emphasize the need for further investigations to determine the potential mechanism of influenza vaccination against stroke in this patient population.

Bui et al. developed a fully automatic AI framework known as DeepHeartCT for multi-structure segmentation in cardiac computed tomography angiography (CTA) images. Their approach effectively addresses the challenge posed by deep learning, which requires a large amount of data and high-quality labels, especially in the realm of medical image analysis. Their AI system was trained and validated on a large clinical cardiac CTA dataset, consisting of over a thousand cases with high-quality computer-generated labels for segmenting various cardiovascular structures. They also proposed a reverse ranking strategy to assess the segmentation quality in the absence of manual reference labels. Their findings underscore the effectiveness of the DeepHeartCT framework in delivering accurate and swift cardiac CTA image segmentation, with potential applicability to large-scale clinical and research applications.

Alshammri et al. performed an extensive study comparing several traditional machine learning (ML) and deep learning (DL) models using voice recordings to detect Parkinson's disease (PD), a common age-related neurological disorder with motor and cognitive symptoms. They also explored advanced feature engineering techniques to enhance model performance. Their results indicate that both ML and DL can be effectively applied for reliable PD prediction. Notably, DL outperformed, achieving an impressive overall accuracy of 98.31%, an overall recall of 98%, an overall precision of 100%, and an f1-score of 99%. These findings hold promise for healthcare applications in PD diagnostics.

Sharma and Verbeke conducted a comprehensive analysis, focusing on various resampling strategies to address the challenge of imbalanced datasets, with a specific case study on diagnosing depression. Their study explores the predictive potential of biomarkers as indicators of depression by leveraging the power of machine learning techniques such as the Extreme Gradient Boosting (XGBoost) algorithm. They applied multiple resampling techniques, encompassing under-sampling, over-sampling, a combination of over-and-under sampling, and the ROSE sampling method, and reported over-sampling is most effective for their application. By exploring the potential of biomarkers in conjunction with machine learning techniques, this research contributes to the growing body of knowledge aimed at improving mental health diagnostics.

Leonard et al. developed a low-dimensional model specifically tailored for patients in the Emergency Department. They explored 15 models, incorporating three machine learning algorithms (Logistic regression, naïve Bayes, and gradient boosting) and five different sampling methods. These models utilize essential patient data, collected up to the post-triage assessment stage. The developed protocol is practical and low-dimensional that can be readily deployed in hospitals that may not have data-rich platforms for models with numerous predictors. Similar to the previous paper, they employed several sampling techniques in dealing with the imbalance between patient admissions and discharges as the outcome variable. This research has the potential to enable early prediction of admissions from the pediatric Emergency Department, potentially improving patient care and resource allocation in healthcare settings.

In conclusion, we express our gratitude to the authors and reviewers who contributed to this Research Topic on Women in AI across the fields of Medicine and Public Health. These high-quality papers showcase the capabilities of female researchers in this field and serve as a powerful reminder of their vital role in advancing knowledge and innovation across various domains, emphasizing the need for increased support to sustain their valuable contributions.

## Author contributions

L-CC: Writing—review and editing. AA: Writing—review and editing.

